# *Clostridium butyricum* extracellular vesicles remodel the transcriptional network of pyroptosis-related genes in LPS-stimulated macrophages

**DOI:** 10.3389/fimmu.2025.1686347

**Published:** 2026-01-05

**Authors:** Qingyu Zhang, Kailun He, Mohd Shafiq Aazmi, Mohd Fakharul Zaman Raja Yahya

**Affiliations:** 1 Faculty of Applied Science, Universiti Teknologi MARA Shah Alam, Shah Alam, Selangor, Malaysia; 2 Department of Food Research, Zhejiang Business College, Hangzhou, Zhejiang, China; 3 Zhejiang Institute for Food and Drug Control, Hangzhou, China; 4 Integrative Pharmacogenomics Institute (iPROMISE), Puncak Alam, Bandar Puncak Alam, Selangor, Malaysia

**Keywords:** *Clostridium butyricum*, extracellular vesicles, pyroptosis, macrophages, bioinformatics

## Abstract

**Background:**

Extracellular vesicles (EVs) of probiotics are an important pathway for probiotics to exert probiotic effects. EVs of *Clostridium butyricum* (*C. butyricum*) has great potential in immunotherapy. The aim of this study is to investigate the regulatory effect of *C. butyricum* EVs on the pyroptosis-related gene network in LPS-stimulated macrophages and explore its potential molecular mechanisms.

**Methods:**

Differential centrifugation was used to isolate *C. butyricum* EVs, and EVs were characterized by transmission electron microscopy, NTA, and ZETA potential. PI staining was employed to confirm the stimulatory effect of LPS on RAW264.7 cells. Transcriptome sequencing and qPCR were performed to validate the regulatory effects of EVs on pyroptosis-related genes (PRGs) in LPS-stimulated RAW264.7 cells.

**Results:**

*C. butyricum* EVs exhibited a bilayer membrane structure with a median particle size of 144.4 ± 85.7 nm and a ZETA potential of -37.56 ± 1.08 mV. PI staining showed that LPS induced cell death in RAW264.7 macrophages, while EV treatment significantly affected the expression of pyroptosis-related genes *IL-18* and *IL-33* (q < 0.05). Transcriptome analysis revealed that *C. butyricum* EVs induce inflammation and cytokine secretion in RAW264.7 macrophages through the JAK-STAT signaling pathway and Cytokine receptor interaction. EVs pretreatment (10/20 μg/mL) regulated the expression of 14 PRGs in a concentration-related manner, including significant upregulation of *IL-6* and *P2rx7* (q < 0.01) and inhibition of *ZBP1* and Tnf (q < 0.05). The gene interaction network showed a positive correlation (r=0.82) between *IL-6* and *P2rx7*, and a negative correlation (r=-0.75) between *ZBP1* and *P2rx7*.

**Conclusion:**

*C. butyricum* EVs remodel the pyroptosis-related regulatory network by modulating key genes such as *IL-6*, *P2rx7*, and *ZBP1*, suggesting their potential role in fine-tuning LPS-induced inflammatory responses in macrophages.

## 1 Introduction

Butyric acid-producing *Clostridium butyricum* (*C. butyricum*) is a type of intestinal probiotic that can regulate the structure of the host’s intestinal microbiota and the body’s immune function, and is of great significance for maintaining intestinal health (1, 2). *C. butyricium* itself has been shown to significantly affect host metabolism and gut microbiota structure (3). Studies have shown that *C. butyricum* can produce extracellular vesicles (EVs), which can regulate the host’s cellular immune function through EVs, regulate intestinal homeostasis and improve colitis (4). While *C. butyricum* EVs have been shown to be non-toxic and have a higher yield than *Lactobacillus rhamnosus* (5), their precise immunomodulatory mechanisms, particularly their impact on the transcriptional programs of immune cells, remain to be fully characterized. One important step is to define the gene regulatory networks influenced by these EVs. Specifically, the transcriptional mechanisms through which *C. butyricum* EVs interact with macrophages to reshape the expression of inflammation- and pyroptosis-related genes are not yet clear. Elucidating this gene-level interaction is essential for understanding their immunoregulatory effects and future therapeutic applications.

Macrophages are the core components of the innate immune system (6), and their functional activation is central to regulating inflammatory responses. A key aspect of macrophage activation is the secretion of pro-inflammatory cytokines such as TNF-α and IL-1β (7). However, excessive immune responses can lead to tissue damage and negatively impact the body. Excessive secretion of inflammatory cytokines can cause pyroptosis, necroptosis, and even cell apoptosis. Studies have shown that *IFN* can induce *ZBP1*, causing inflammatory cell death in macrophages (8). Inflammatory cell death of macrophages, especially pyroptosis, plays an important role in the occurrence of various diseases (9, 10).

Studies have shown that the extracellular vesicles of intestinal commensal bacteria can regulate cellular inflammatory responses, affect the expression of inflammatory cytokines, and also affect LPS induced inflammatory responses (11–13). Some intestinal bacterial extracellular vesicles weaken LPS induced NLRP3 inflammasome activation by affecting the *NLRP3-Caspase 1-ASC* signaling pathway, thereby reducing inflammatory responses (14). Apoptosis is closely related to inflammatory response, and *NLRP3* inflammasome is an important link in cell apoptosis (15). Regulating the *NLRP3*/*Caspase 1* signaling pathway is an effective method to inhibit cell apoptosis (16). EVs from beneficial intestinal bacteria, such as *C. butyricum*, may possess the capacity to influence these processes. Preliminary evidence suggests that *C. butyricum* EVs can regulate inflammatory cytokines and mitigate LPS-induced inflammatory responses, warranting further investigation into their potential role in modulating inflammatory cell death. This study aims to explore the potential mechanism of the immunomodulatory effect of *C. butyricum* EV on RAW264.7 macrophages, and investigate their regulatory impact on the pyroptosis-related gene network in LPS-stimulated RAW264.7 macrophages.

## 2 Materials and methods

### 2.1 Materials

The following materials were utilized: RAW264.7 macrophages (CTCC-001-0048) sourced from Zhejiang Meisen Cell Technology Co., Ltd. (China); *C. butyricum* strain CBM588 procured from Miyarisan (Japan); The *C. butyricum* CBM588 (MIYAIRI 588) is a well-documented and commercially used probiotic strain, employed in commercial pharmaceutical preparations such as MIYA-BM TABLETS. Its selection is based on its established safety profile and proven efficacy in modulating host biology, which lends significant relevance to our investigation into host-inflammatory responses. Reinforced Clostridial Medium (RCM) from Haibo Biotechnology Co., Ltd. (China); and Eagle’s Minimum Essential Medium (EMEM, 1X) from Gibco (USA). Fetal bovine serum (FBS; heat-inactivated) and lipopolysaccharide (LPS) were supplied by Shanghai Datsil Bio-Tech Co., Ltd. (China) and Beyotime (China), respectively. Molecular reagents included a reverse transcription kit (Vazyme, China), AceQ qPCR SYBR Green Master Mix (Vazyme, China), and a total RNA extraction kit (TIANGEN, China). DEPC-treated water was acquired from Sangon Biotech (China). DL-Dithiothreitol (DTT) and Tris were purchased from Solarbio (China). Trypsin was obtained from Promega (USA). Iodoacetamide (IAM) was sourced from Aladdin (China). Phenylmethanesulfonyl fluoride (PMSF) was bought from Xiya Reagent (China). Tetraethylammonium bromide (TEAB) and Urea were procured from Sigma (USA). Ethylene Diamine Tetraacetic Acid (EDTA), Xylene brilliant cyaninG (G250), Sodium dodecyl sulfate (SDS), Thiourea, and acetone were supplied by Sinopharm (China). PCR primers and the BCA Protein Assay Kit were obtained from Beyotime (China). Pyridine iodide (PI) ws obtained from Biosharp (Beijing, China).

Instrumentation encompassed a Nano particle tracking analyzer (ZetaView, Particle Metrix, Germany) equipped with software version 8.05.14 SP7, ultracentrifuges (Avanti JXN26 and Optima XPN100, Beckman, USA), and an FEI Tecnai transmission electron microscope (USA). Consumables such as fluorescence quantitative PCR plates, polystyrene microspheres, and filter membranes were procured from Axygen (USA), Applied-Microspheres (Germany), and Merck Millipore (Ireland), respectively. The mass spectrometer (Orbitrap™ Astral™) was purchased from Thermo Fisher Scientific (USA). The ultrasonic cell disruptor (JY92-11N) was sourced from SCIENTZ (China). The benchtop high-speed refrigerated centrifuge (TGL-20M) was obtained from CENCE (China). The vacuum freeze dryer (CV 600) was acquired from Beijing JM Technology Co., Ltd (China). The electrophoresis power supply (DYY-6C) and electrophoresis tank (DYCZ-24DN) were purchased from LIUYI (China). The microplate reader (Cmax Plus) was obtained from Molecular Devices (USA).

### 2.2 Bacterial culture protocol

*C. butyricum* colonies were initially streaked onto solid Reinforced Clostridium Medium (RCM) and incubated anaerobically at 37 °C overnight. A single colony was transferred to liquid RCM medium for 24–36 hr under anaerobic conditions. Subsequently, 1%–2% (v/v) of this culture was inoculated into 2 L MCP liquid medium and incubated anaerobically at 37 °C for 24 hr. The bacterial suspension was adjusted to OD600 = 0.5 using fresh RCM medium and further incubated anaerobically at 37 °C for 24 hr prior to extracellular vesicle (EV) extraction.

### 2.3 EVs isolation and characterization

EVs were purified from *C. butyricum* cultures following Morishita et al. (5). Briefly, 100 mL supernatant was sequentially centrifuged (4,000g, 10 min; 10,000g, 30 min; 4 °C) to remove cellular debris, filtered through a 0.45 μm membrane, and ultracentrifuged (100,000g, 70 min; 4 °C). Pelleted EVs were washed twice with PBS via ultracentrifugation (100,000g, 70 min; 4 °C) and quantified via Bradford assay.

Transmission electron microscopy (TEM) was utilized to corroborate the morphological integrity of the isolated EVs. A small volume (15 μL) of the EVs suspension was adsorbed onto a carbon-coated copper grid for one minute. Any residual liquid was subsequently removed using filter paper. Negative staining was carried out by applying 15 μL of a 2% (v/v) uranyl acetate solution to the grid for one minute, after which the stain was blotted off. The grids were air-dried completely prior to imaging. TEM observations were conducted to assess the ultrastructural features of the EVs.

Nanoparticle tracking analysis (NTA) was employed to determine the size distribution and concentration of *C. butyricum* EVs. Following purification, EVs samples were diluted to a concentration of approximately 5 × 10^7^ particles/mL. For each measurement, 500 μL of the diluted sample was introduced into the nanoparticle tracking analyzer. All samples were analyzed by capturing data from five randomly selected visual fields. The instrument settings were configured as follows: camera sensitivity was set to 16, the recording duration per video was 60 seconds, and the detection threshold was fixed at 7. Remaining parameters were maintained at their default values. Data analysis was performed using the instrument’s native software (version 8.04.02). Zeta potential was also determined via nanoparticle tracking analysis (ZetaView) with five fields analyzed per sample.

All EVs preparations were subjected to sterility testing by incubation in bacterial culture medium at 37 °C for 48 hours prior to cellular treatments. Only batches confirmed to be free of microbial contamination were used in subsequent experiments.

### 2.4 Macrophage culture, treatment, and assessment of pyroptosis

RAW264.7 cells were seeded in 12-well plates at a density of 1×10^6^ cells per well and maintained in F12K medium supplemented with 10% fetal bovine serum (FBS), 100 U/mL penicillin-streptomycin, and 5% CO_2_ at 37 °C. Five experimental groups were established (**Table 1**). After the respective treatments, cellular morphological changes in all groups were directly observed and recorded using an inverted phase-contrast microscope, with particular attention to pyroptosis-related features such as cell swelling and membrane blebbing.

**Table 1:** Treatment of the cells.

Group	Phase 1 treatment	Note
1	PBS (12 h) → PBS (12 h)	
2	EVs (10 μg/mL, 12 h) → PBS (12 hr)	EVs 10 μg/mL = 3.28×10^10^ Particle/mL
3	PBS (12 h) → LPS (1 μg/mL, 12 h)	
4	EVs (10 μg/mL, 12 h) → LPS (1 μg/mL, 12 h)	EVs 10 μg/mL = 3.28×10^10^ Particle/mL
5	EVs (20 μg/mL, 12 h) → LPS (1 μg/mL, 12 h)	EVs 20 μg/mL = 6.55×10^10^ Particle/mL

To assess plasma membrane integrity, Propidium Iodide (PI) staining was performed. The cell culture supernatant from each treatment group was collected. Adherent cells were gently washed once with phosphate-buffered saline (PBS). The supernatant and the wash PBS were combined and centrifuged to pellet any detached cells. The adherent cells were then trypsinized and pooled with the pelleted, detached cells. The combined cell pellet was washed twice with cold PBS and resuspended. A PI staining solution was added to the cell suspension at a final concentration of 0.5 mg/mL, followed by incubation at 4 °C in the dark for 15 minutes. After staining, the cells were immediately observed and imaged using a fluorescence microscope with excitation and emission wavelengths of approximately 488 nm and 630 nm, respectively. Cells exhibiting red fluorescence were considered PI-positive, indicating a loss of plasma membrane integrity.

### 2.5 Total RNA extraction, cDNA construction and transcriptome analysis

RNA extraction followed the manufacturer’s protocol using a Total RNA Kit I. Cells were lysed with Trizol, and RNA was purified through chloroform extraction and isopropanol precipitation. Purified RNA was dissolved in DEPC-treated water. cDNA synthesis was conducted following a denaturation step at 65 °C for 5 minutes, reverse transcription at 42 °C for 30 minutes, and enzyme inactivation at 70 °C for 15 minutes.

Transcriptome libraries were sequenced on an Illumina NovaSeq 6000 platform at Shanghai Biozeron Co., Ltd (Shanghai, China). Use Trimmatic software to filter out adapters and low-quality reads (reads with a length less than 35 NT, containing n bases, and a Q value<20) to obtain high-quality clean data. Then, the transcriptome was assembled by *de novo* splicing using Trinity software, and redundant sequences were removed using rseqc (RNA SEQ data quality control) software to obtain the UniGene of the transcriptome. UniGene with a length exceeding 200 bp will be compared and annotated in multiple public databases. For example, nucleic acid sequence database (NT), conserved domain database (CDD), non-redundant protein database (NR), protein family alignment and hidden Markov model database (PFAM), and homologous protein cluster database (COG).

### 2.6 q-PCR

Gene expression analysis of target genes was performed using q-PCR on a CFX96 real-time system (Bio-Rad, USA). Primer sequences and reaction conditions are detailed in **Tables 2**–**4**.

**Table 2:** q-PCR reaction system.

Reagent	Consumption
cDNA	2 μL
2×SYBR Green Mix	5 μL
Forward Primer(10μM)	0.5 μL
Reverse Primer(10μM)	0.5 μL
RNase free ddH20	2 μL
Total Volume	10 μL

**Table 3:** q-PCR reaction conditions.

Reaction period	Temperature	Time
Pre-change period	95°C	30s
	94°C	10s
Cycle period	60°C	20s
	94°C	30s
Dissolution curve analysis	55°C	1min
	94°C	30s

**Table 4:** Sequences of primers.

Gene names	Primer-F (5′-3′)	Primer-R (5′-3′)	Length (bp)
GAPDH	AAGAAGGTGGTGAAGCAGG	GAAGGTGGAAGAGTGGGAGT	111
Tnf	TTAGAAAGGGGATTATGGCTCA	TTTGCAGAACTCAGGAATGGAC	182
Pelp1	AAGATGTGGAGTTTGGTTCAGC	CAATAAGAGCCCAGGTTCAGG	134
Nlrp6	CCTCCAGGCAAGTCTTTCAA	CCAACCGCAGAGTGTCCAG	118
Tgfb2	CCCGAATAAAAGCGAAGAGC	GGAGGGCAACAACATTAGCAG	148
Gsdma	GGAGCAAAATTGGAGCTATGA	CATCCTGAGTTGAGGGTTGAA	148
Gsdmc2	ACCTGGAGGCTAACTTGAATGT	TTGGGATGCTTGTACTCTGAAC	102
Gsdmc4	TCCTGGATGAGCTGCGAAAG	GGAGCCCCATCTTCACAGAC	138
Gzmn	GGTGAATCCAGGGGATGTG	AGCCTCTATTTTGTTTGGGTCT	187
Pjvk	AAAATGGGTTCGTTGCCTGA	GGCTTGGGACAGGGACTAAT	124
P2rx7	CAGCGGAAAGAGCCTGTTAT	CCTGCAAAGGGAAGGTGTAGTC	142
Nlrp1a	CAGAGGCTCAGTGGTTAGCAG	AGTGGGAAGGAGGCTGTTG	124
Timp2	TCGCCGTGAGCGTTCTTG	GCATTCCACTCTGGGTTCG	121
IL-6	CACAGAAGGAGTGGCTAAGGA	GCACTAGGTTTGCCGAGTAGAT	101
IL-1β	GGGCTGGACTGTTTCTAATGC	CTTGTGACCCTGAGCGACC	128
IL-18	AAGGACACTTTCTTGCTTGCC	ACAAACCCTCCCCACCTAAC	134
IL-33	GAGTGGCGTTACCTGTGCTT	CGGGTGGGAACTAAAATGTCT	125
IL12	ATTTTGAAAGGCTGGGTATCG	TGTGCTGGAACTCCCTCTGTA	118
ZBP1	AAGGCTGCTGTGAGCATGG	CGGTAAAGGACTTGATTGAGGG	167

### 2.7 Bioinformatics analysis

The bioinformatics analysis of the transcriptomic data was performed using a series of computational tools and pipelines (17). To explore the role of pyroptosis-related genes (PRGs), we integrated our DEGs with a list of 121 PRGs previously reported in the literature (17–19). The gene difference analysis between groups were analyzed using edgeR software, with significant conditions of abs (logFC)>=1 and padj<0.05. According to the intersection genes (the intersection genes of differential genes and pyroptosis genes), the calculation of differential genes in sample grouping was conducted using limma software to explore the expression differences between high and low expression genes. A Pvalue less than 0.05 and | logFC |>0.5 were considered as differences. Use the clusterProfiler package to enrich upregulated differentially expressed genes and downregulated differentially expressed genes separately, using algorithms ORA and GSEA, with the database used for ORA being Gene Ontology (GO), Encyclopedia of Genes and Genomes (KEGG), Reactome. The functional databases used by GSEA are HALLMRARK, GOBP, and KEGG, and the signatures of the functions come from the MSIGDB database. Visualize the enrichment results using the enrichplot package.

### 2.8 Data analysis

All experiments were repeated three times and all data were presented as mean ± standard deviation (n=3). Statistical significance was assessed using independent t-tests, with p<0.05 considered significant.

## 3 Results

### 3.1 Characteristics of *C. butyricum*-derived EVs

**Figure 1:**
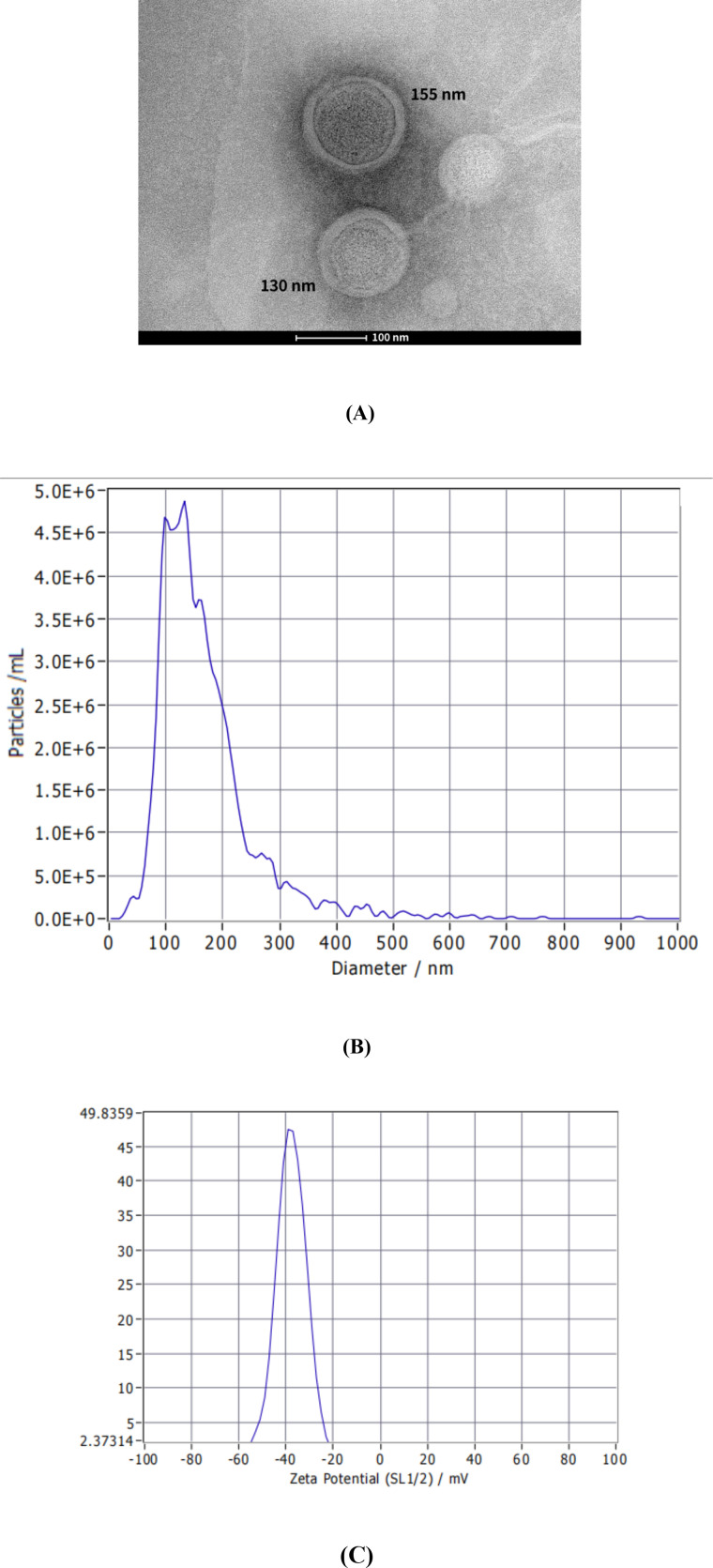
Characteristics of *C*. *butyricum* EVs. **(A)** TEM image of *C*. *butyricum*-derived EVs, **(B)** NTA result, **(C)** Zeta Potential result.

After centrifugation, a pale yellow precipitate was found at the bottom of the centrifuge tube, which was completely dissolved after adding sterile water. Transmission Electron Microscope images showed that *C. butyricum*-derived EVs exhibited a distinct double-layered vesicle structure (**Figure 1A**, consistent with previous research reports (4). Nanoparticle tracking analysis (NTA) indicated that the median particle size of *C. butyricum* EVs was 144.4 ± 85.7 nm (**Figure 1B**), with a particle concentration of 3.9 × 10^11^ particles/mL. Zeta Potential measurements showed that the zeta potential of *C. butyricum* EVs was -37.56 ± 1.08 mV (**Figure 1C**). The protein concentration of the EVs preparation, as determined by the BCA assay, was 119 μg/mL. Consequently, the particle-to-protein ratio was calculated to be approximately 3.28 × 10^9^ particles/μg.

### 3.2 Observation of cell morphology and evaluation of cell death by PI staining

To evaluate the cell death status induced by LPS, this study conducted inverted phase contrast microscopy morphological observation and propidium iodide (PI) fluorescence staining detection on five experimental groups.

**Figure 2:**
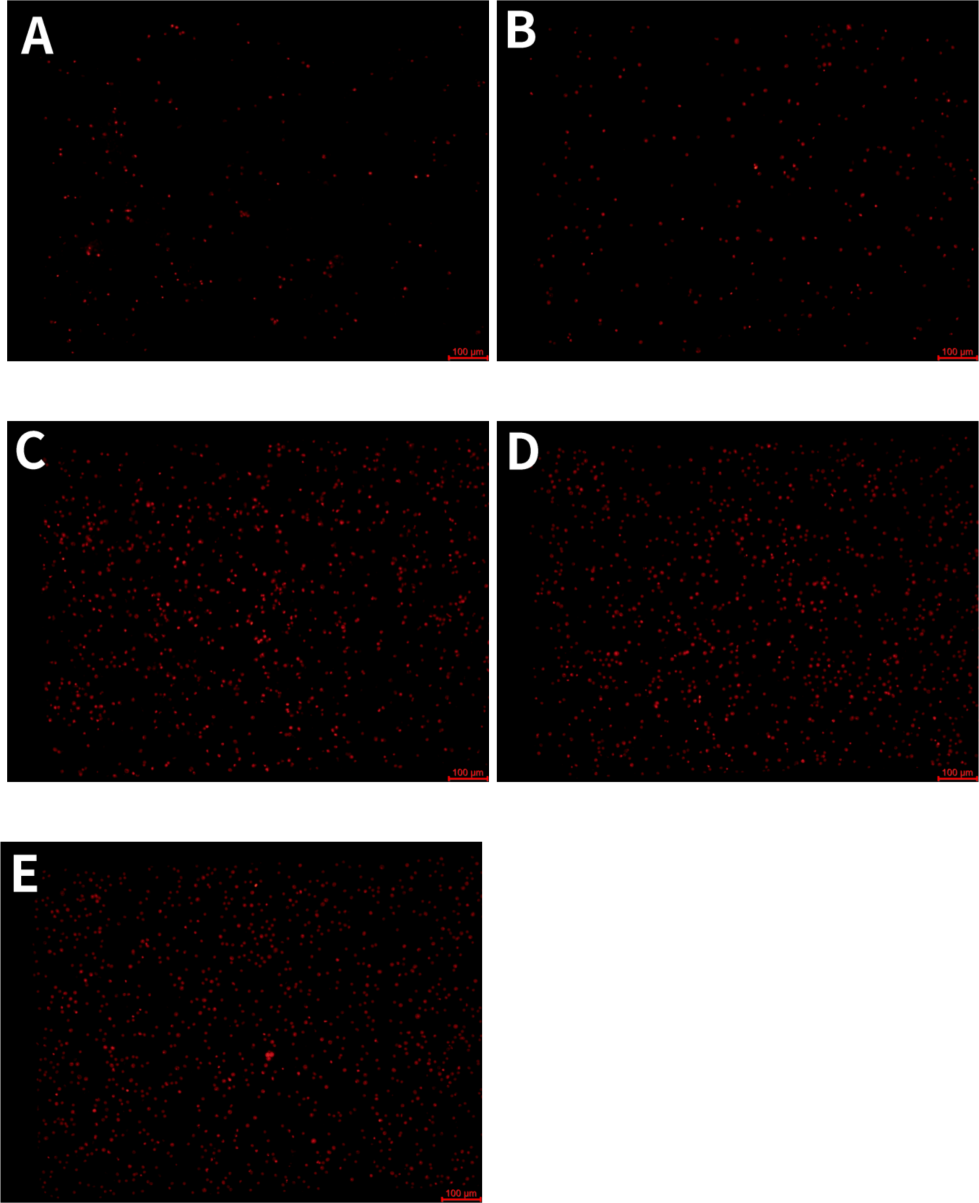
RAW264.7 macrophage PI cell staining. **(A)** Control, **(B)** EVs only, **(C)** LPS only, **(D)** EVs (10 μg/mL, 12 h) + LPS (1 μg/mL, 12 h), **(E)** EVs (20 μg/mL, 12 h) + LPS (1 μg/mL, 12 h).

The PI staining results (**Figure 2**) showed that compared with the negative control group and the simple EVs treatment group, the LPS treatment group exhibited high-intensity PI fluorescence signals, indicating that LPS stimulation led to significant loss of membrane integrity in RAW264.7 cells. However, no significant differences were observed in PI fluorescence intensity between the LPS treatment group, the EVs (10 μg/mL) + LPS treatment group, and EVs (20 μg/mL) + LPS treatment group.

In terms of cell morphology (**Supplementary Figure S1**), under an inverted phase contrast microscope, it was observed that the cells in the LPS treated group exhibited significant morphological features similar to pyroptosis, such as swelling and membrane rupture. Similar phenomena were also observed in the EVs+LPS treatment group. It is worth noting that in the pure EVs treatment group, although some changes in cell morphology can be observed, the PI fluorescence signal intensity is much lower than that of the LPS treatment group and its combined treatment group.

These results indicate that LPS treatment can induce significant cell death in RAW264.7 cells, but the specific mode of death and the role of *C. butyricum* EVs in this process need further validation.

### 3.3 Inflammatory cytokines and pyroptosis-related genes

**Figure 3:**
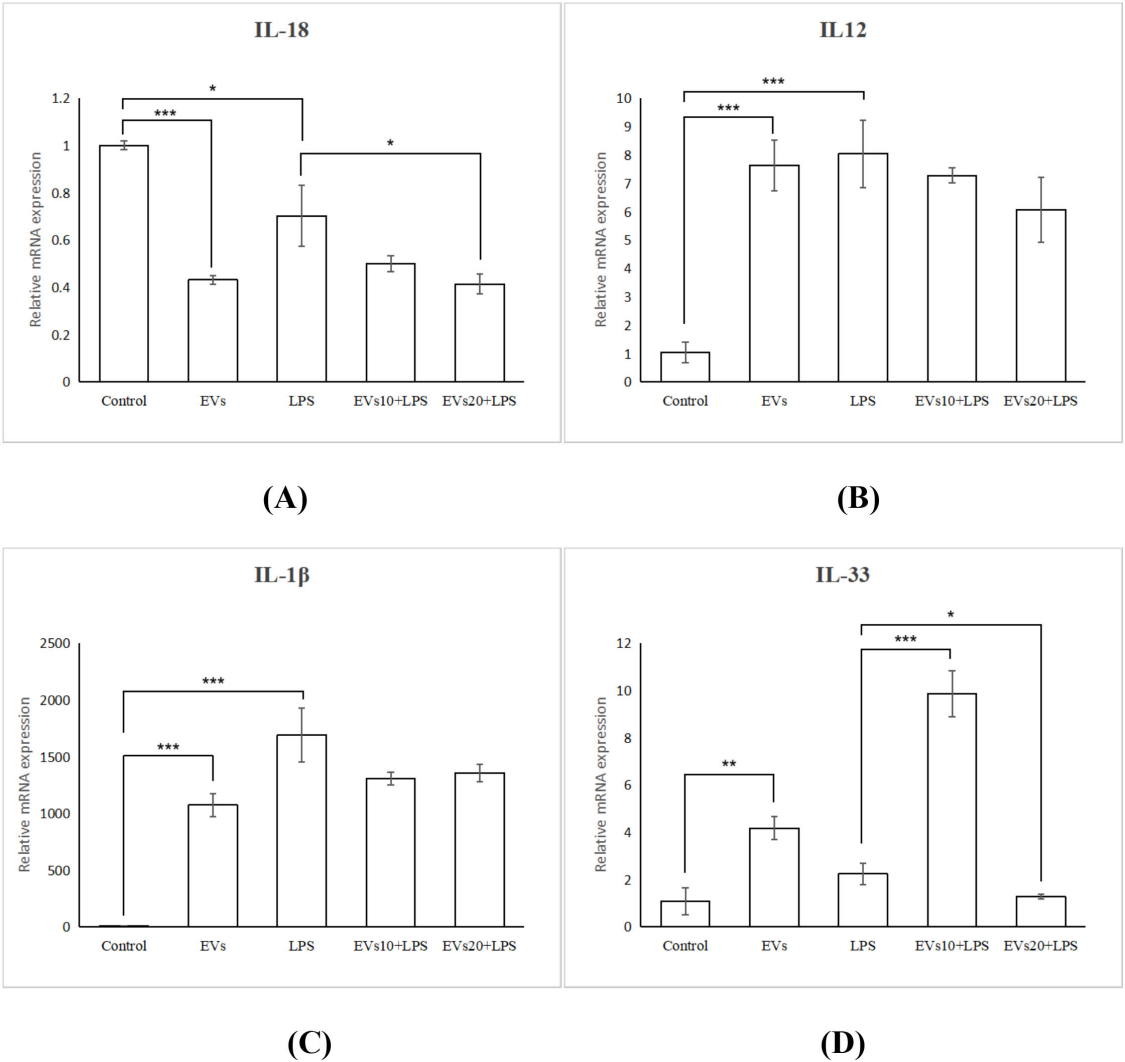
Inflammatory cytokines and pyroptosis markers mRNA expression in RAW264.7 macrophages. **(A)** IL-18, **(B)** IL-12, **(C)** IL-1β, **(D)** IL-33. *P < 0.05, **P < 0.01, ***P < 0.0001.

We detected the expression of four inflammatory cytokine genes, *IL-1β*, *IL*-*12*, *IL*-*18*, and *IL*-*33* (**Figure 3**), among which *IL-1β*, *IL-18*, and I*L-33* are pyroptosis-related genes (20, 21). The results showed that *C. butyricum* EVs modulated the LPS-induced expression of these genes, including a non-significant reduction in *IL-1β* (q>0.05) and a significant inhibition of *IL-18* (q<0.05). Under the treatment of 10 μg/mL EVs, the expression of IL-33 induced by LPS significantly increased (q<0.001), while under the treatment of 20 μg/mL EVs, it was significantly decreased (q<0.05). Treatment with *C. butyricum* EVs also reduced the expression of *IL-12* induced by LPS, but the results were not significant.

### 3.4 Identification of differentially expressed genes and key pathways in RAW264.7 cells treated with EVs

**Figure 4:**
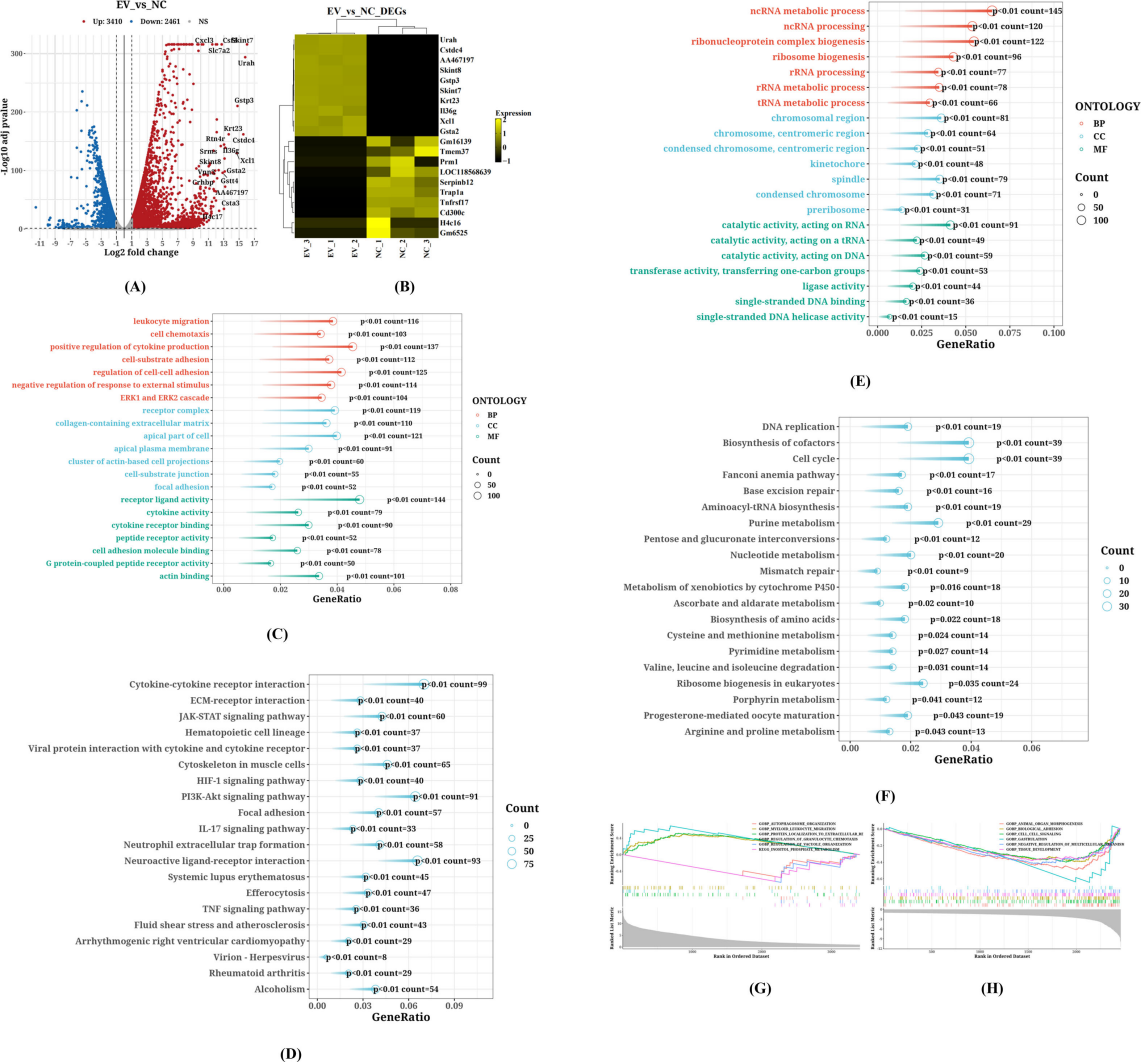
Identification of DEGs and Key Pathways in RAW264.7 Cells Treated with EVs. **(A)** The volcano plot of DEGs, **(B)** The heatmap of DEGs, **(C)** The KEGG pathways enriched by upregulated genes, **(D)** The GO annotations enriched by upregulated genes, **(E)** The KEGG pathways enriched by downregulated genes, **(F)** The GO annotations enriched by downregulated genes, **(G)** The GSEA enrichment analysis results for upregulated genes, **(H)** The GSEA enrichment analysis results for downregulated genes.

We compared the transcriptome of cells treated with 10 μg/mL *C. butyricum* EVs with that of the control group. According to **Figures 4A, B**, a total of 5871 DEGs were identified (P<0.05, | logFC |>1), with 3410 genes upregulated and 2461 genes downregulated. **Figures 4C, D** illustrate the KEGG pathways and GO annotations enriched by upregulated genes, while **Figures 4E, F** depict those for downregulated genes.

The enrichment analysis revealed that the upregulated gene pathways primarily involve “Cytokine-cytokine receptor interaction,” “ECM-receptor interaction,” “JAK-STAT signaling pathway,” “Hematopoietic cell lineage,” and “Viral protein interaction with cytokine and cytokine receptor.” In contrast, the major downregulated pathways include “Alcoholism,” “DNA replication,” “Biosynthesis of cofactors,” “Cell cycle,” “Fanconi anemia pathway,” “Base excision repair,” “Aminoacyl-tRNA biosynthesis,” and “Purine metabolism.”

In the biological processes (BP) category of GO annotation results, upregulated genes were predominantly enriched in “leukocyte migration,” “cell chemotaxis,” “positive regulation of cytokine production,” “cell-substrate adhesion,” “regulation of cell-cell adhesion,” “negative regulation of response to external stimulus,” and “ERK1 and ERK2 cascade.” Downregulated genes showed significant enrichment in “ncRNA metabolic process,” “ncRNA processing,” “ribonucleoprotein complex biogenesis,” “ribosome biogenesis,” “rRNA processing,” “rRNA metabolic process,” and “tRNA metabolic process.”

Regarding cellular components (CC) in GO annotations, the top five significantly enriched terms for upregulated genes included “receptor complex,” “collagen-containing extracellular matrix,” “apical part of cell,” “apical plasma membrane,” and “cluster of actin-based cell projections.” For downregulated genes, the top five significantly enriched terms were “chromosomal region,” “chromosome,” “centromeric region,” “condensed chromosome,” and “kinetochore” or “spindle.”

In the molecular function (MF) category of GO annotations, the top five significantly enriched terms for upregulated genes comprised “receptor ligand activity,” “cytokine activity,” “cytokine receptor binding,” “peptide receptor activity,” and “cell adhesion molecule binding.” Meanwhile, “catalytic activity acting on RNA,” “catalytic activity acting on a tRNA,” “catalytic activity acting on DNA,” “transferase activity transferring one-carbon groups,” and “ligase activity” were the most significantly enriched terms for downregulated genes.

**Figures 4G, H** represent the GSEA enrichment analysis results for upregulated and downregulated genes, respectively. According to the GSEA analysis, the functions of differentially expressed genes mainly encompass “inositol phosphate metabolism” (Enrichment Score: -0.6442, P Value: 0.0000), “regulation of vacuole organization” (Enrichment Score: -0.6694, P Value: 0.0001), “autophagosome organization” (Enrichment Score: -0.5536, P Value: 0.0002), “protein localization to extracellular region” (Enrichment Score: 0.5170, P Value: 0.0002), and “regulation of granulocyte chemotaxis” (Enrichment Score: 0.6939, P Value: 0.0003).

The results indicated that after treatment with *C. butyricum* EVs, the upregulated genes in RAW264.7 cells were significantly enriched in immune-modulating pathways such as “Cytokine-cytokine receptor interaction” (e.g., IL-6, TNF signaling), “JAK-STAT signaling pathway,” and “ECM-receptor interaction.” The MF of these genes were concentrated in “receptor ligand activity” and “cytokine activity,” while BP involved “leukocyte migration,” “cell chemotaxis,” and “ERK1/2 signaling cascade.” In contrast, downregulated genes primarily participated in fundamental metabolic processes including “DNA replication,” “cell cycle,” and “purine metabolism,” and were enriched in chromosome-associated structures within CC (such as “chromosomal region,” “kinetochore”). Further GSEA analysis revealed that EVs treatment significantly inhibited pathways like “phosphatidylinositol metabolism” and “vacuole organization” (P<0.001), while activating “protein extracellular localization” and “regulation of granulocyte chemotaxis”. These findings suggest that C. butyricum EVs modulate macrophage inflammatory responses and functional homeostasis by reshaping immune-related gene networks (such as cytokine signaling) and suppressing cell proliferation-related pathways.

### 3.5 Identification of DEGs and key pathways in LPS-stimulated RAW264.7 cells treated with EVs

**Figure 5:**
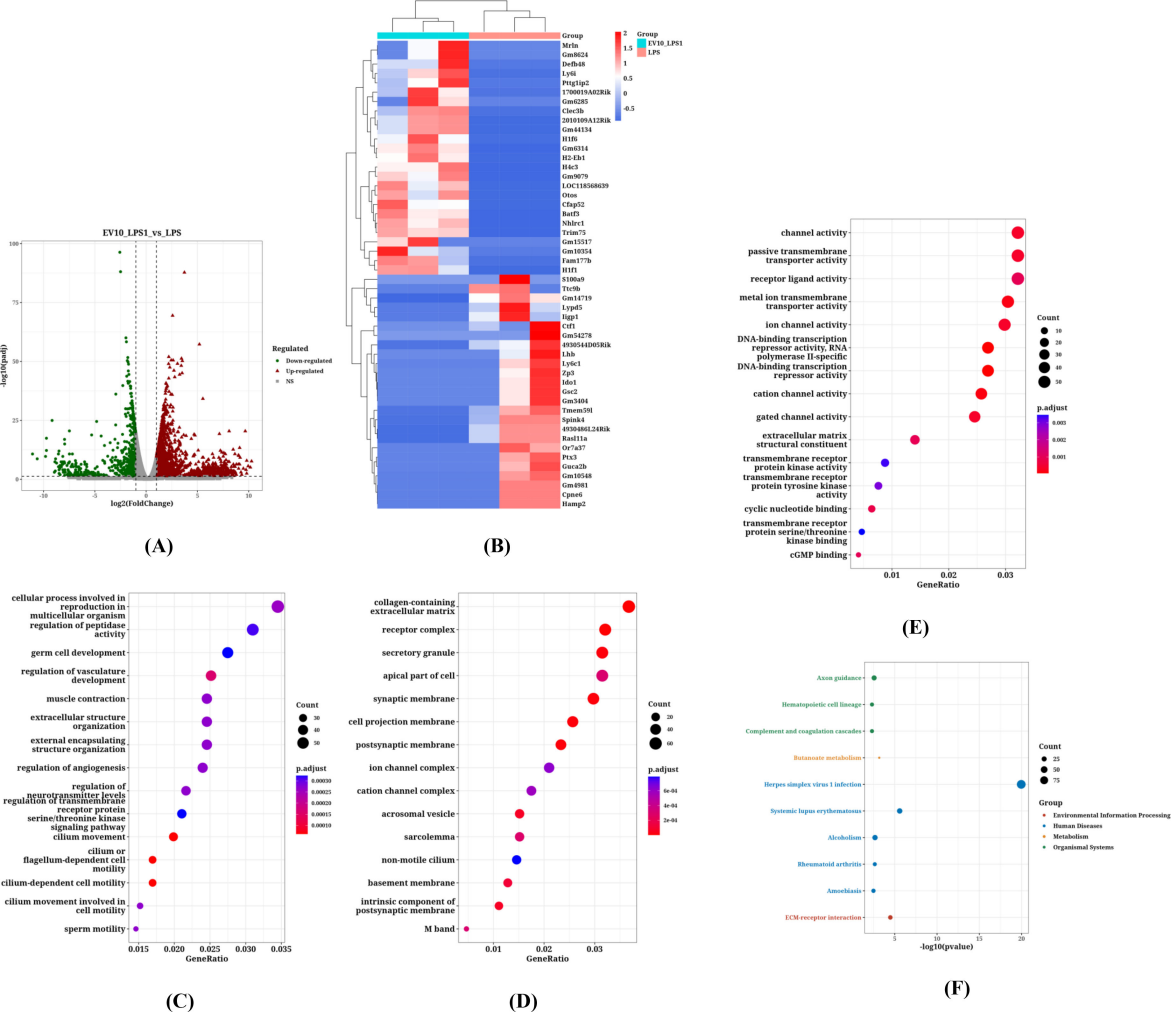
The Impact of Pre-treatment with 10 μg/mL *C*. *butyricum* EVs on the Transcriptome of Macrophages Following LPS Stimulation. **(A)** The volcano plot of DEGs, **(B)** The heatmap of DEGs, **(C)** The Biological Process (BP) categories of GO for upregulated genes, **(D)** The Cellular Component (CC) categories of GO for upregulated genes, **(E)** The Molecular Function (MF) categories of GO for upregulated genes, **(F)** The KEGG pathways enriched by upregulated genes.

We pre-treated LPS-stimulated RAW264.7 macrophages with *C. butyricum* EVs at concentrations of 10 μg/mL and 20 μg/mL, respectively. By comparing the transcriptomes of cells treated with 10 μg/mL of *C. butyricum* EVs to those of the control group, 2648 DEGs were identified based on the screening criteria (P < 0.05 and |logFC| > 1), of which 2007 genes were upregulated and 641 genes were downregulated (**Figures 5A, B**). **Figures 5C–E** present the ORA enrichment analysis results for the BP, CC, and MF categories of GO for upregulated genes. **Figure 3F** displays the KEGG pathway analysis results for upregulated genes.

According to the GO annotation results, the upregulated DEGs were primarily enriched in BP such as “cellular process involved in reproduction in multicellular organisms,” “regulation of peptidase activity,” “germ cell development,” “regulation of vasculature development,” “muscle contraction,” and “extracellular structure organization.” In the CC category, significantly enriched terms included “collagen-containing extracellular matrix,” “receptor complex,” “secretory granule,” “apical part of cell,” “synaptic membrane,” and “cell projection membrane.” Notably, in the MF category, the upregulated genes were significantly enriched in terms like “channel activity,” “passive transmembrane transporter activity,” “receptor ligand activity,” “metal ion transmembrane transporter activity,” and “ion channel activity.”

The KEGG pathway analysis highlighted several important pathways, including “Herpes simplex virus 1 infection,” “Systemic lupus erythematosus,” “Alcoholism,” “Axon guidance,” “ECM-receptor interaction,” and “Amoebiasis.”

**Figure 6:**
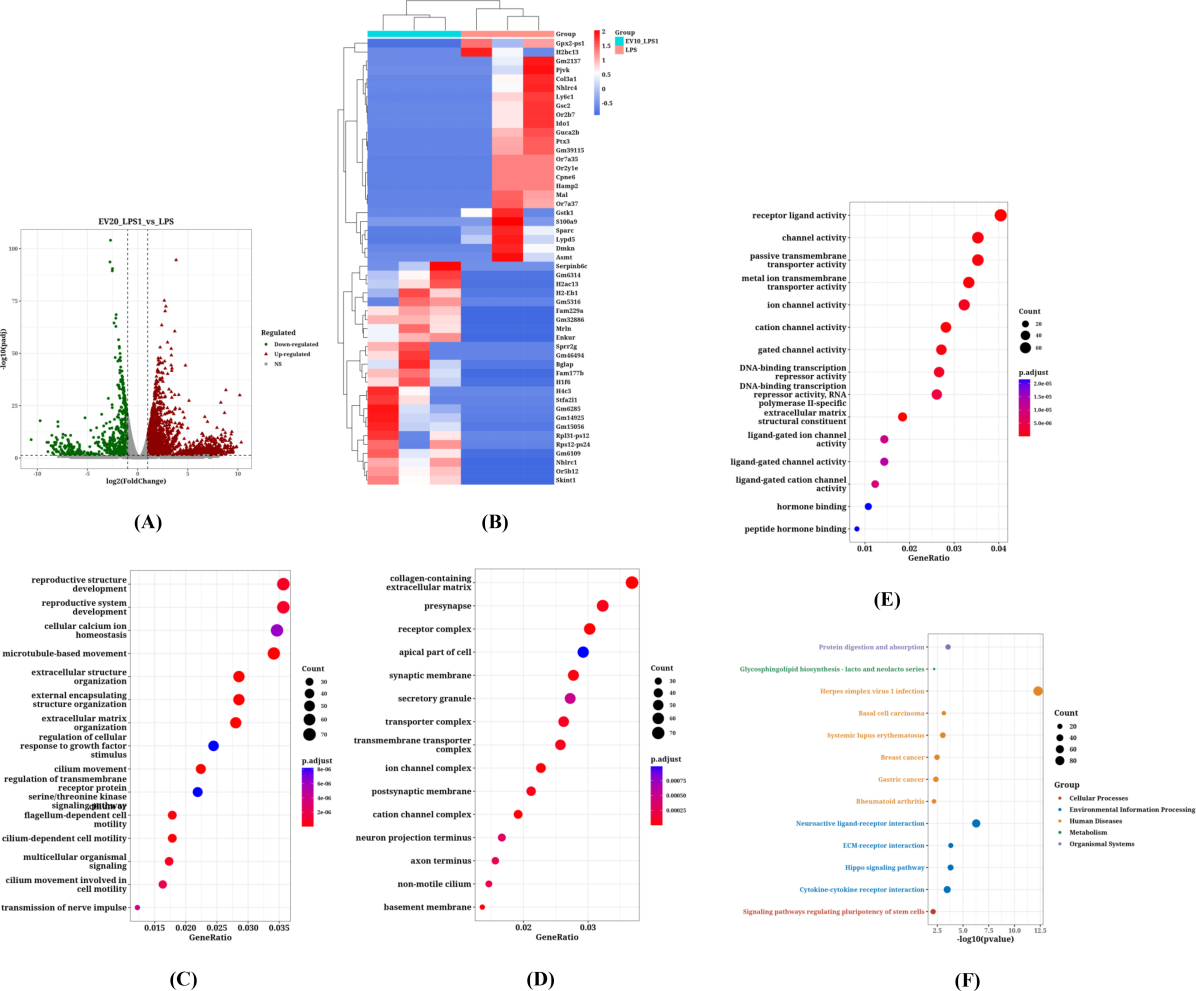
The Impact of Pre-treatment with 20 μg/mL *C*. *butyricum* EVs on the Transcriptome of Macrophages Following LPS Stimulation. **(A)** The volcano plot of DEGs, **(B)** The heatmap of DEGs, **(C)** The Biological Process (BP) categories of GO for upregulated genes, **(D)** The Cellular Component (CC) categories of GO for upregulated genes, **(E)** The Molecular Function (MF) categories of GO for upregulated genes, **(F)** The KEGG pathways enriched by upregulated genes.

The results of pre-treatment with 20 μg/mL of *C. butyricum* EVs were similar to those obtained with 10 μg/mL *C. butyricum* EVs. By comparing the transcriptomes of cells treated with 20 μg/mL of *C. butyricum* EVs to those of the control group, a total of 2950 DEGs were identified, among which 2280 genes were upregulated and 670 genes were downregulated. The volcano plot and heatmap of these DEGs are shown in **Figures 6A, B**, respectively. **Figures 6C–E** present the ORA enrichment analysis results for the BP, CC, and MF categories of GO for upregulated genes. **Figure 4F** displays the KEGG pathway analysis results for upregulated genes.

### 3.6 Expression patterns of differentially expressed PRGs in RAW264.7 cells

**Figure 7:**
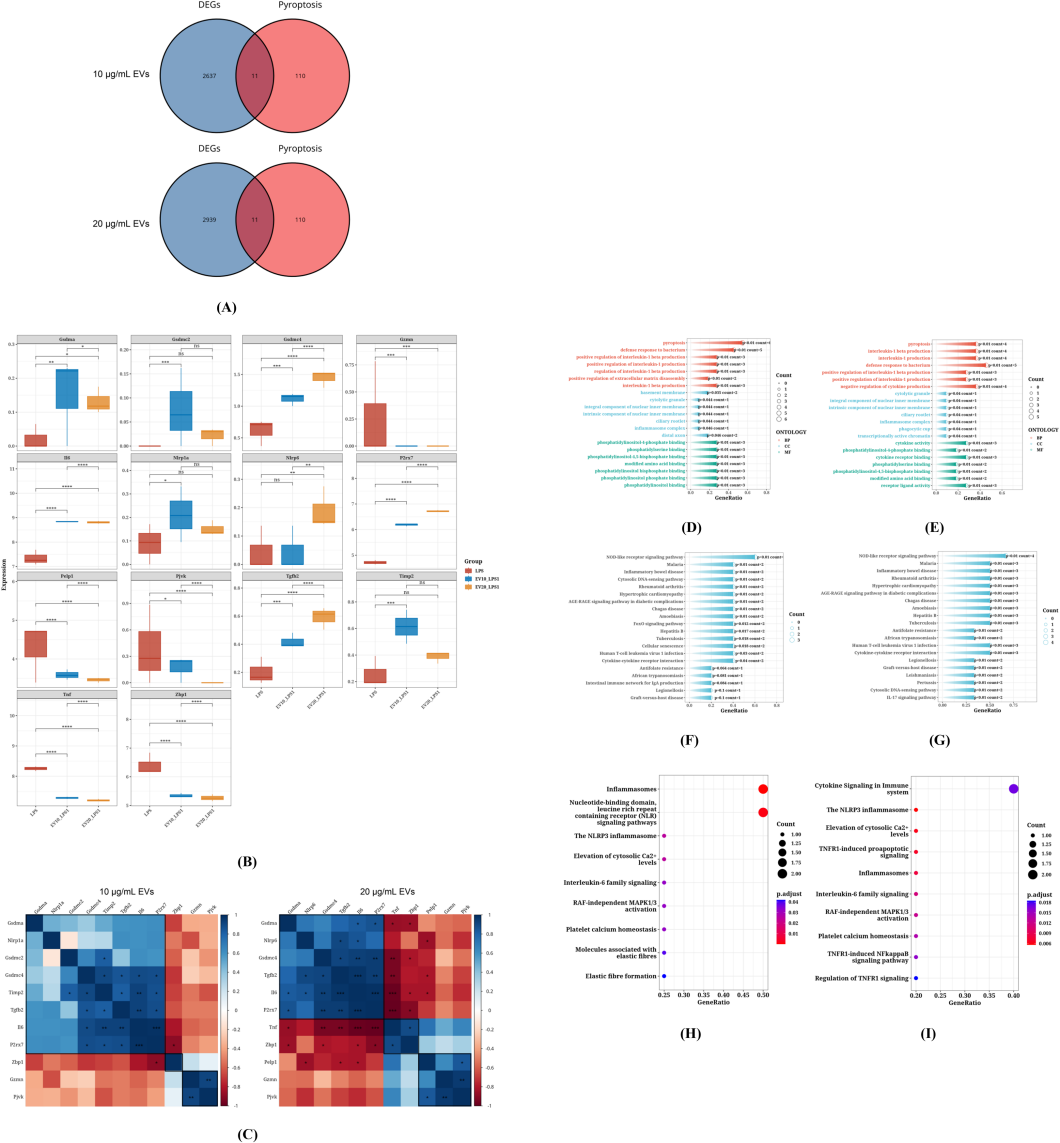
Expression Patterns of Differentially Expressed PRGs in RAW264.7 Cells. **(A)** The Venn diagram of DEGs and Pyroptosis, **(B)** Differential expression of PRGs, **(C)** The heatmap of PRGs gene expression correlation, **(D)** GO enrichment analysis of PRGs following treatment with 10 μg/mL *C*. *butyricum* EVs, **(E)** GO enrichment analysis of PRGs following treatment with 20 μg/mL *C*. *butyricum* EVs, **(F)** KEGG pathway enrichment analysis of PRGs following treatment with 10 μg/mL *C*. *butyricum* EVs, **(G)** KEGG pathway enrichment analysis of PRGs following treatment with 20 μg/mL *C. butyricum* EVs, **(H)** Enrichment analysis results of PRGs in the Reactome database following treatment with 10 μg/mL *C*. *butyricum* EVs, **(I)** Enrichment analysis results of PRGs in the Reactome database following treatment with 20 μg/mL *C. butyricum* EVs.

The qPCR results showed that *C. butyricum* EVs could regulate the pyroptosis-related genes *IL-1β*, *IL-33* and *IL-18*. To further understand the potential mechanism by which *C. butyricum* EVs modulate the pyroptosis-related gene network, we analyzed the expression patterns of differentially expressed PRGs in RAW264.7 cells. We conducted an intersection analysis between a panel of PRGs and the DEGs identified from transcriptome sequencing after EVs pre-treatment (**Figure 7A**). A total of 14 differentially expressed PRGs were identified. The PRGs following treatment with 10 μg/mL *C. butyricum* EVs included *Gsdma*, *Gsdmc2*, *Gsdmc4*, *Gzmn*, *IL-6*, *Pjvk*, *ZBP1*, *P2rx7*, *Nlrp1a*,T*gfb2*, and *Timp2*. The PRGs following treatment with 20 μg/mL *C. butyricum* EVs included *Gsdma*, *Gsdmc4*, *Gzmn*, *IL-6*, *Nlrp6*, *Pjvk*, *Tnf*, *Zbp1*, *P2rx7*, *Pelp1*, and *Tgfb2*. **Figure 7B** illustrates the expression differences of these 14 PRGs between the EVs groups and the LPS control group. Among them, *Gsdma*, *Gsdmc4*, *IL-6*, *Nlrp6*, *P2rx7*, *Pelp1*, and *Timp2* were found to be upregulated in the EVs-treated groups, while *Gsdmc2*, *Gzmn*, *Nlrp1a*, *Pjvk*, *Tnf*, *Zbp1*, and *Tgfb2* were downregulated. Moreover, the expression changes of these PRGs were concentration-responsive, with the extent of upregulation or downregulation being significantly altered at the higher EV concentration (20 µg/mL) compared to the lower dose (10 µg/mL). The heatmap in **Figure 7C** represents the correlation of expression levels among the intersecting genes. Correlation analysis indicated that among the PRGs, *ZBP1* showed the strongest negative correlation with *P2rx7*, whereas *P2rx7* exhibited the strongest positive correlation with *IL-6*, *IL-6* with *Tgfb2*, and *IL-6* with *Timp2*.

GO enrichment analysis suggested that these PRGs are primarily involved in “pyroptosis,” “defense response to bacterium,” “positive regulation of interleukin-1 beta production,” and “positive regulation of interleukin-1 production” (**Figures 7D, E**). KEGG pathway enrichment analysis indicated that these PRGs are mainly associated with the “NOD-like receptor signaling pathway,” “Malaria,” “Inflammatory bowel disease,” and “Intestinal immune network for IgA production” (**Figures 7F, G**). Additionally, **Figures 5H, I** show the enrichment analysis results for the intersecting genes in the Reactome database, indicating that PRGs are most highly enriched in “Inflammasomes,” “Nucleotide-binding domain,” and “leucine-rich repeat-containing receptor (NLR) signaling pathways.” Collectively, these bioinformatic analyses suggest that the regulatory effects of *C. butyricum* EVs are functionally linked to core pyroptosis and inflammatory pathways. These findings are consistent with a concentration-dependent remodeling of the pyroptosis-related transcriptional network by *C. butyricum* EVs, wherein the shift from 10 to 20 µg/mL differentially modulated the expression of key genes, including *IL-6*, *P2rx7*, and *ZBP1*.

### 3.7 Biological process analysis of PRGs

**Figure 8:**
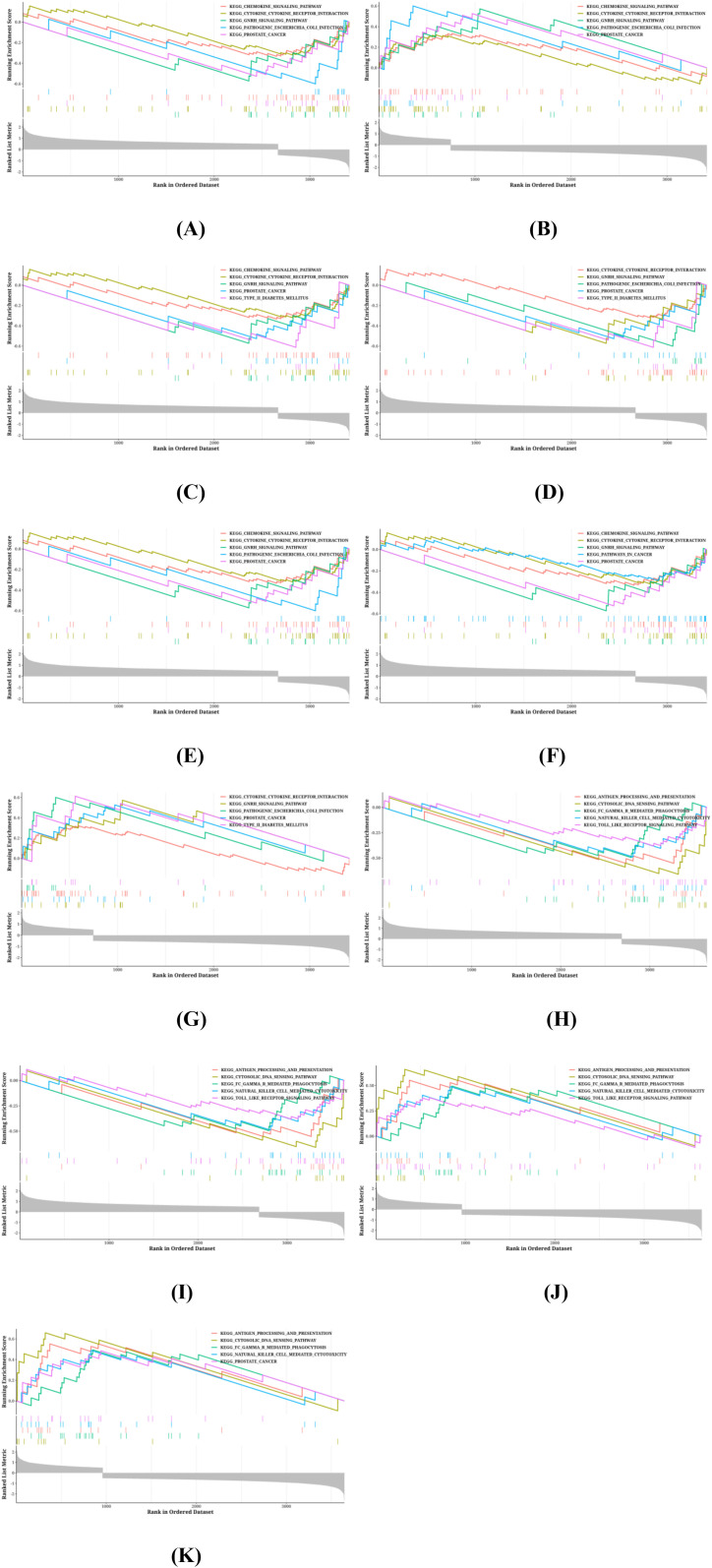
Related biological process of differentially expressed PRGs. **(A)** Gsdmc4, **(B)** Gzmn, **(C)** IL-6, **(D)** P2rx7, **(E)** Tgfb2, **(F)** Timp2, **(G)** ZBP1, **(H)** Gsdma, **(I)** Nlrp6, **(J)** Pjvk, **(K)** Tnf.

To gain deeper insights into the pathways associated with the identified PRGs, GSEA analysis was conducted on the 14 PRGs. Among them, 7 genes (*Gsdmc4*, *Gzmn*, *IL-6*, *P2rx7*, *Tgfb2*, *Timp2*, and *ZBP1*) were analyzed in the 10 μ g/mL *C. butyricum* EVs treatment group. Ten genes (*Gsdma*, *Gsdmc4*, *Gzmn*, *IL-6*, *Nlrp6*, *Pjvk*, *Tnf*, *Zbp1*, *P2rx7*, and *Tgfb2*) were analyzed from the 20 μg/mL *C. butyricum* EVs treatment group, while *Pelp1*, *Gsdmc2* and *Nlrp1a* did not obtain GSEA results. **Figure 8** shows the top five GSEA results of the KEGG pathway based on these genes, with *Gsdmc4*, *Gzmn*, *IL-6*, *P2rx7*, *Tgfb2*, *Timp2*, and *ZBP1* from the 10 μ g/mL *C. butyricum* EVs treatment group, and *Gsdma*, *Nlrp6*, *Pjvk*, and *Tnf* from the 20 μ g/mL *C. butyricum* EVs treatment group. According to the GSEA analysis, the primary functions related to these PRGs include the “GNRH signaling pathway” (Enrichment Score: 0.5728, P Value: 0.0000), “Prostate cancer” (Enrichment Score: 0.5268, P Value: 0.0002), “Cytokine-cytokine receptor interaction” (Enrichment Score: 0.3200, P Value: 0.0002), “Pathogenic Escherichia coli infection” (Enrichment Score: 0.6012, P Value: 0.0003), and “Type II diabetes mellitus” (Enrichment Score: 0.6124, P Value: 0.0003).

### 3.8 qPCR validation of PRGs

**Figure 9:**
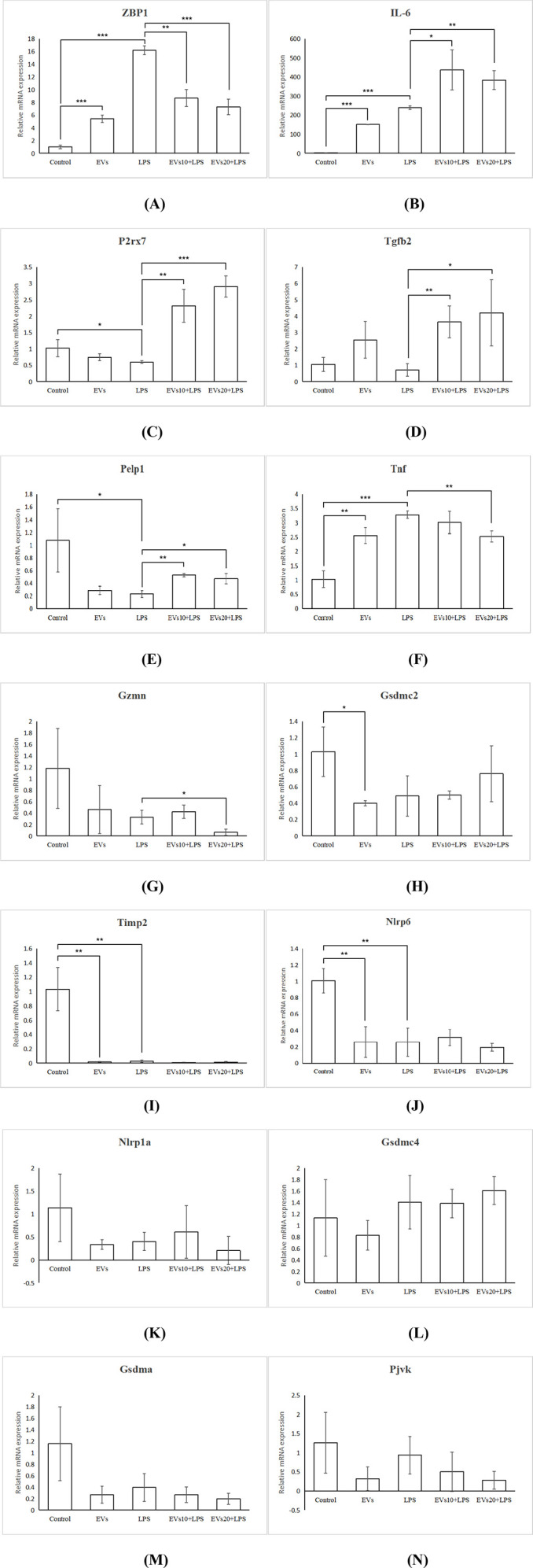
PRGs mRNA expression in RAW264.7 macrophages. **(A)** ZBP1, **(B)** IL-6, **(C)** P2rx7, **(D)** Tgfb2, **(E)** Pelp1, **(F)** Tnf, **(G)** Gzmn, **(H)** Gsdmc2, **(I)** Timp2, **(J)** Nlrp6, **(K)** Nlrp1a, **(L)** Gsdmc4, **(M)** Gsdma, **(N)** Pjvk. *P < 0.05, **P < 0.01, ***P < 0.0001.

Quantitative PCR (qPCR) was employed to validate the expression of 14 differentially expressed PRGs (*Gsdma*, *Gsdmc2*, *Gsdmc4*, *Gzmn*, *IL-6*, *Pjvk*, *ZBP1*, *P2rx7*, *Nlrp1a*, *Tgfb2*, *Timp2*, *Nlrp6*, *Pelp1*, *Tnf*) in LPS-induced RAW264.7 macrophage models following EVs treatment. **Figure 9** demonstrated that EVs significantly inhibited the expression of *ZBP1* (q < 0.001), *Gzmn* (q < 0.05) and *Tnf* (q<0.01). Moreover, EVs significantly promoted the expression of *IL-6* (q < 0.01), *P2rx7* (q < 0.001), *Tgfb2* (q < 0.01) and *Pelp1*(q<0.01). Additionally, EVs appeared to inhibit the expression of *Gsdma*, *Timp2*, and *Pjvk*, as well as promote the expression of *Gsdmc4*, *Gsdmc2* and *Nlrp6*, however these effects were not statistically significant (q > 0.05).

## 4 Discussion

This study elucidates the modulatory effects of *C. butyricum* EVs on the transcriptional network of PRGs and associated signaling pathways in LPS-stimulated RAW264.7 macrophages. Through transcriptome analysis and qPCR validation, we characterized the physicochemical properties of the EVs, delineated their impact on the macrophage transcriptome, and provided a theoretical foundation for understanding the gene-regulatory potential of probiotic-derived EVs in the context of inflammatory responses.

Research has shown that extracellular vesicles are an important pathway for the interaction between gut microbiota and host cells. Bacterial extracellular vesicles can transmit various information molecules to host cells, triggering immune responses (22). This study used nanoparticle tracking analysis (NTA) to show that the median particle size of EVs was 144.4 ± 85.7 nm, with a ZETA potential of -37.56 ± 1.08 mV, which was similar to that of previous studies (5). The particle size distribution was smaller than that of EVs derived from *Lactobacillus casei* (202.9 ± 88.9 nm) (23). The particle-to-protein ratio of our EV preparation was calculated to be 3.28 × 10^9^ particles/μg. A ratio below 1.5 × 10^9^ particles/μg is typically considered indicative of impurity (Whittaker et al., 2020). Therefore, our value suggests a relatively high purity of the isolated EVs, though it also implies the potential co-isolation of a minor amount of non-vesicular protein.

Studies have shown that *C. butyricum* can regulate the host’s cellular immune function through EVs, regulate intestinal homeostasis and improve colitis (4, 24). We found that *C. butyricum* EVs modulated the expression of inflammatory cytokines in RAW264.7 macrophages, exhibiting distinct effects under basal conditions and upon LPS stimulation. Transcriptome analysis showed that EVs treatment alone significantly upregulated the JAK-STAT signaling pathway in RAW264.7 cells. Research has shown a direct correlation between the JAK-STAT pathway and inflammatory response (25, 26). The JAK-STAT pathway is the main downstream signaling pathway for key inflammatory factors such as *IL-6*. After *IL-6* binds to receptors, it activates *STAT3* through *JAK1*/2, which can promote the expression of inflammatory cytokines (27). QPCR validation confirmed that treatment of RAW264.7 cells with *C. butyricum* EVs upregulated the expression level of *IL-6*, which may contribute to the observed upregulation of the JAK-STAT signaling pathway. Under LPS stimulation, EVs pretreatment further upregulated the expression of *IL-6* (q<0.01), while inhibiting the expression of *Tnf* (q<0.01). This pattern of gene regulation is consistent with previous studies indicating that *IL-6* can suppress LPS-induced *Tnf* expression, suggesting a potential anti-inflammatory mechanism (28).

Pyroptosis is an inflammatory programmed cell death (29). During the process of cell pyroptosis, it is generally accompanied by the formation of cell membrane pores, membrane rupture, cell swelling, and release of cellular contents. The occurrence of cell pyroptosis is closely related to inflammatory cytokines, and has become a hot topic in programmed cell death (30). Cellular pyroptosis often interacts with apoptosis and necroptosis, leading to inflammatory cell death. Together, the three are synthesized into PANoptosis (31). During the process of cell pyroptosis, the cell membrane ruptures and cell contents are released through *GSDM* mediation, thus cell pyroptosis is also defined as *GSDM* mediated programmed cell death (32). The GSDM family (GSDMA, GSDMB, GSDMC, GSDMD, GSDME, PVJK/DFNB59, etc.) is the executor of cell pyroptosis (33). Most of the proteins in the *GSDM* family can form oligomers, insert into the cell membrane or mitochondrial membrane, form pores, cause cell rupture and pyroptosis, and release IL-1 β, IL-18, and DAMPs, thereby triggering a strong inflammatory response (34). It is generally believed that *Gsdma* is related to cellular mitochondrial homeostasis (35), but recent studies have shown that *Gsdma* can also mediate cell apoptosis. Studies have shown that severe starvation can induce cell pyroptosis through phosphorylation induced *Gsdma* (36). African swine fever virus (ASFV) can activate caspase-3 and caspase-4, cleave GSDMA to regulate cell pyroptosis (37), and *SpeB* from group A Streptococcus can cleave and activate *Gsdma* to mediate cell pyroptosis (38). *Gsdmc* can mediate cell pyroptosis, and the caspase-8/GSDMC pathway is an important pathway for cell pyroptosis. GSDMC is specifically cleaved by caspase-8 under TNF - α treatment, producing a GSDMC-N-terminal domain that forms pores on the cell membrane and induces pyroptosis (39, 40). *Gsdmc2* and *Gsdmc4* are mainly expressed in the stomach, large intestine, small intestine, bladder, and prostate of mice (32). Research has found that overexpression of *Gsdmc2* in HEK293 cells can trigger cell pyroptosis and lytic cell death (41). *Pjvk* is also a member of the GSDM protein family, but it does not have the ability to punch holes on the cell membrane, and its mechanism of action in cell pyroptosis has not been reported (42). Our study found that after treating LPS induced RAW264.7 macrophages with *C. butyricum* EVs, although the expression of *Gsdmc2* and *Gsdmc4* was upregulated and the expression of *Gsdma* and *Pjvk* was downregulated. However, the expression levels of these genes were low and the observed changes were not statistically significant (q>0.05). Therefore, the regulatory contribution of these particular GSDM family members to the overall transcriptional network alterations induced by *C. butyricum* EVs appears to be limited.

*P2rx7*(P2X7R) is believed to have the function of an inflammatory regulator, which can affect the regulation of the body’s immune system, influence the occurrence of inflammation and cancer development, and is one of the key genes in the body’s immune regulation (43). P2X7R is an adenosine triphosphate (ATP) gated ion channel that can be activated by ATP and BzATP, among others. The P2X7R-NEK7-NLRP3 axis is an important pathway that triggers cell pyroptosis. Research has shown that P2X7R mediated potassium efflux can induce NLRP3 inflammasome assembly and activation, thereby inducing pyroptosis of prostate epithelial cells (44). During LPS induced apoptosis of mouse liver cells, LPS activates NLRP3 through P2X7R to activate macrophages and induce hepatocyte pyroptosis (45). Meanwhile, studies have shown that the ATP mediated P2X7R paracrine mechanism is a P2X7 receptor dependent membrane repair mechanism. P2X7R achieves isolation and detachment of damaged plasma membrane segments by inducing Ca2+influx, foaming the cell membrane, and protecting the cell body from further damage by limiting elevated intracellular Ca2+. Studies have shown that the expression of P2X7R can enhance the resistance of HEK cells to bacterial pore forming toxin attacks (46). Transcriptome analysis and qPCR results showed that *C. butyricum* EVs significantly increased the expression of *P2rx7* in LPS induced RAW264.7 macrophages (q<0.001). This upregulation suggests that the effect of EVs is not mediated by suppressing *P2rx7*. Given the dual role of P2X7R in promoting pyroptosis and membrane repair, the functional consequence of its increased expression warrants further investigation to determine if it contributes to a pro-pyroptotic signal or a protective membrane repair response in this context.

*Pelp1* is a scaffold protein that can act as a co regulatory factor for multiple transcription factors and nuclear receptors (47). Recent studies have found that *Pelp1* also plays an important role in cell pyroptosis, and inhibiting the expression of *Pelp1* can reduce cell pyroptosis. For example, miR-195-5p alleviates pyroptosis in GC-1 cells by inhibiting *Pelp1* expression during OGD/R, and this protective effect is blocked when miR-195-5p is downregulated (48). Metformin induces GSDMD mediated pyroptosis in esophageal squamous cell carcinoma cells by targeting the miR-497/*Pelp1* axis (49). We found through qPCR that *C. butyricum* EVs significantly increased the expression of *Pelp1* in LPS induced RAW264.7 macrophages (q<0.001). This upregulation suggests that the modulation of the pyroptosis-related gene network by *C. butyricum* EVs is not mediated through the suppression of *Pelp1* expression.

ZBP1 is an important protein that regulates cell death and plays a regulatory role in cell pyroptosis and necroptosis (50, 51). *ZBP1* is an important regulatory factor for cell pyroptosis, and studies have found that *ZBP1* can induce pyroptosis in acute liver injury through the PGAM5/ROS pathway cluster (52). *ZBP1* is an important cell apoptosis regulator in the occurrence and progression of systemic lupus erythematosus (53). *ZBP1* activates NLRP3 inflammasome through the RIPK1-RIPK3-caspase-8 axis (54), and ZBP1-NLRP3 inflammasome promotes the maturation of pro-inflammatory cytokines and GSDMD by activating caspase-1. GSDMD is cleaved by caspase-1, forming a pore on the cell membrane that releases cytokines and causes cell pyroptosis (55). Gzmn is a granule enzyme that induce cell apoptosis and pyroptosis, but the specific mechanism is not yet clear. Research has found that GZMA can cleave GSDMB, causing pores in the cell membrane and leading to cell pyroptosis (56). Our study found that *C. butyricum* EVs significantly inhibited the expression of *ZBP1* and *Gzmin* in LPS induced RAW264.7 macrophages (q<0.05). The downregulation of these key pyroptosis-related genes suggests that *ZBP1* and *Gzmn* may represent important nodes in the transcriptional network through which *C. butyricum* EVs modulate pyroptosis-related signaling.

Research has shown that bacterial EVs can predict diseases, regulate host immune responses, and serve as drug delivery carriers (57). The production of *C. butyricum* EVs is relatively high, almost free of toxic substances, and has great potential for medical applications. *C. butyricum* EVs can regulate the structure of mouse gut microbiota, regulate intestinal homeostasis, and improve acute colitis (4). *C. butyricum* EVs can also alleviate LPS induced acute lung injury (58). However, due to the influence of bacterial culture conditions on the types and characteristics of bacterial EVs’ contents, the medical ability of bacterial EVs varies under different culture conditions (59). Therefore, the stability of the efficacy of bacterial EVs is an important factor limiting their application. Our study elucidates the transcriptional regulatory effects of *C. butyricum* EVs on pyroptosis-related genes in RAW264.7 macrophages *in vitro*, which has significant limitations.

While this study provides novel insights into the transcriptional regulatory effects of *C. butyricum* EVs, it is important to acknowledge its limitations. The primary limitation lies in the lack of direct functional evidence to confirm the execution of pyroptosis, such as measuring GSDMD cleavage or LDH release. Furthermore, the characterization of EVs purity and the confirmation of membrane-enclosed cargo were limited by the lack of specific assays, such as Western blot analysis for EVs-specific markers and protease/nuclease protection assays. Additionally, the particle-to-protein ratio observed in our EVs preparations is consistent with the potential co-isolation of non-vesicular components, a recognized limitation of the differential centrifugation method employed. An additional limitation is the narrow concentration range of EVs (10 and 20 µg/mL) tested, which, while showing bioactivity, precludes a definitive dose-response analysis and the identification of a potential cytotoxic threshold. Consequently, the optimal effective concentration range of *C. butyricum* EVs remains to be fully elucidated. At the same time, the discovery of mouse macrophage lineage models needs to be validated in more complex *in vivo* systems. Future research should focus on employing more stringent EVs purification methods, incorporating the aforementioned functional assays to validate pyroptosis, and performing a comprehensive dose-response assessment using a broader concentration gradient (e.g., up to 50 µg/mL) to determine the IC50 and assess potential cytotoxicity. Ultimately evaluating the therapeutic potential of these EVs in relevant animal models of inflammatory disease.

## 5 Conclusion

This study successfully isolated and identified extracellular vesicles (EVs) of *C. butyricum*, with a median particle size of 144.4 ± 85.7 nm and a ZETA potential of -37.56 ± 1.08 mV. Transcriptome analysis revealed that EVs treatment significantly regulated 3410 genes in RAW264.7 macrophages, mainly enriched in signaling pathways such as JAK-STAT signaling pathway and Cytokine-cytokine receptor interaction. EVs pretreatment significantly regulated 2648 genes in LPS-stimulated RAW264.7 macrophages, and 14 PRGs (*Gsdma*, *Gsdmc2*, *Gsdmc4*, *Gzmn*, *IL-6*, *Pjvk*, *ZBP1*, *P2rx7*, *Nlrp1a*, *Tgfb2*, *Timp2*, *Nlrp6*, *Pelp1*, *Tnf*) were identified among them. Among them, in the 10 μg/mL EVs treatment group, the expression of *IL-6*, *P2rx7*, *Tgfb2*, and *Timp2* was upregulated (q<0.01), while the expression of *ZBP1*, *Gzmn*, *Tnf*, and *Gsdmc2* was inhibited (q<0.05); The 20 μg/mL EVs treatment group further upregulated *NLRP6* and inhibited *Pjvk* (q<0.01). Gene interaction analysis showed that *IL-6* was significantly positively correlated with *P2rx7*(r=0.82), while *ZBP1* was negatively correlated with *P2rx7* (r=-0.75). These data indicate that *C. butyricum* EVs modulate inflammatory and cytokine-related pathways in RAW264.7 macrophages through pathways such as JAK-STAT signaling pathway and Cytokine-cytokine receptor interaction and remodel the expression network of key pyroptosis-related genes. This transcriptional reprogramming suggests a potential role for *C. butyricum* EVs in fine-tuning the macrophage response to LPS stimulation. This study provides novel insights into the gene-regulatory function of probiotic-derived EVs.

## Data Availability

The datasets presented in this study can be found in online repositories. The names of the repository/repositories and accession number(s) can be found below: PRJNA1308677 (SRA).
