# Supplementary material for: Clostridium butyricum extracellular vesicles remodel the transcriptional network of pyroptosis-related genes in LPS-stimulated macrophages
Source: Front Immunol. 2026 Jan 5;16:1686347. doi: 10.3389/fimmu.2025.1686347 (PMC12812611; doi:10.3389/fimmu.2025.1686347)
Supplement: Supplementary file 1 [file DataSheet1.docx]

**Supplementary Figure**


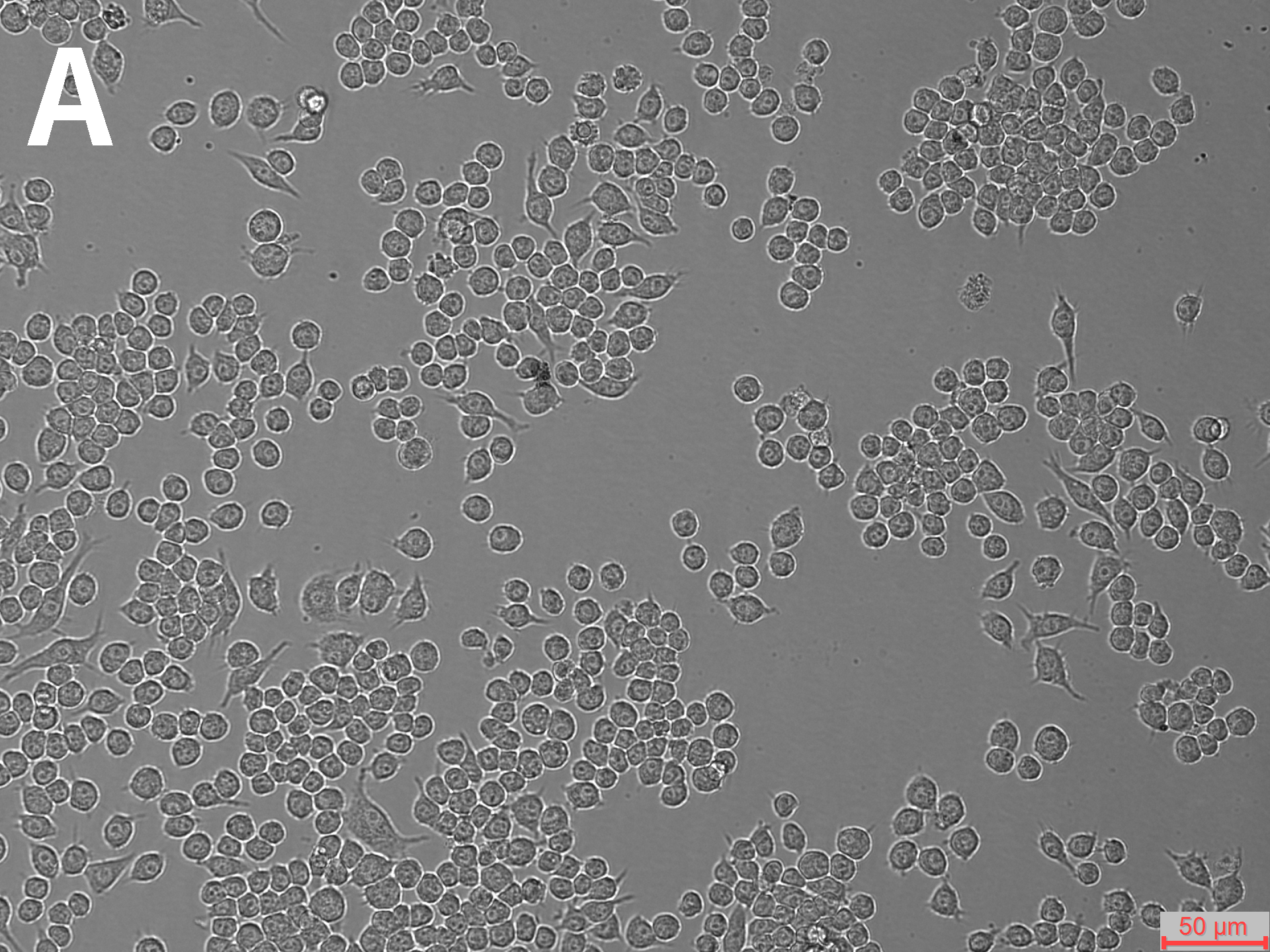

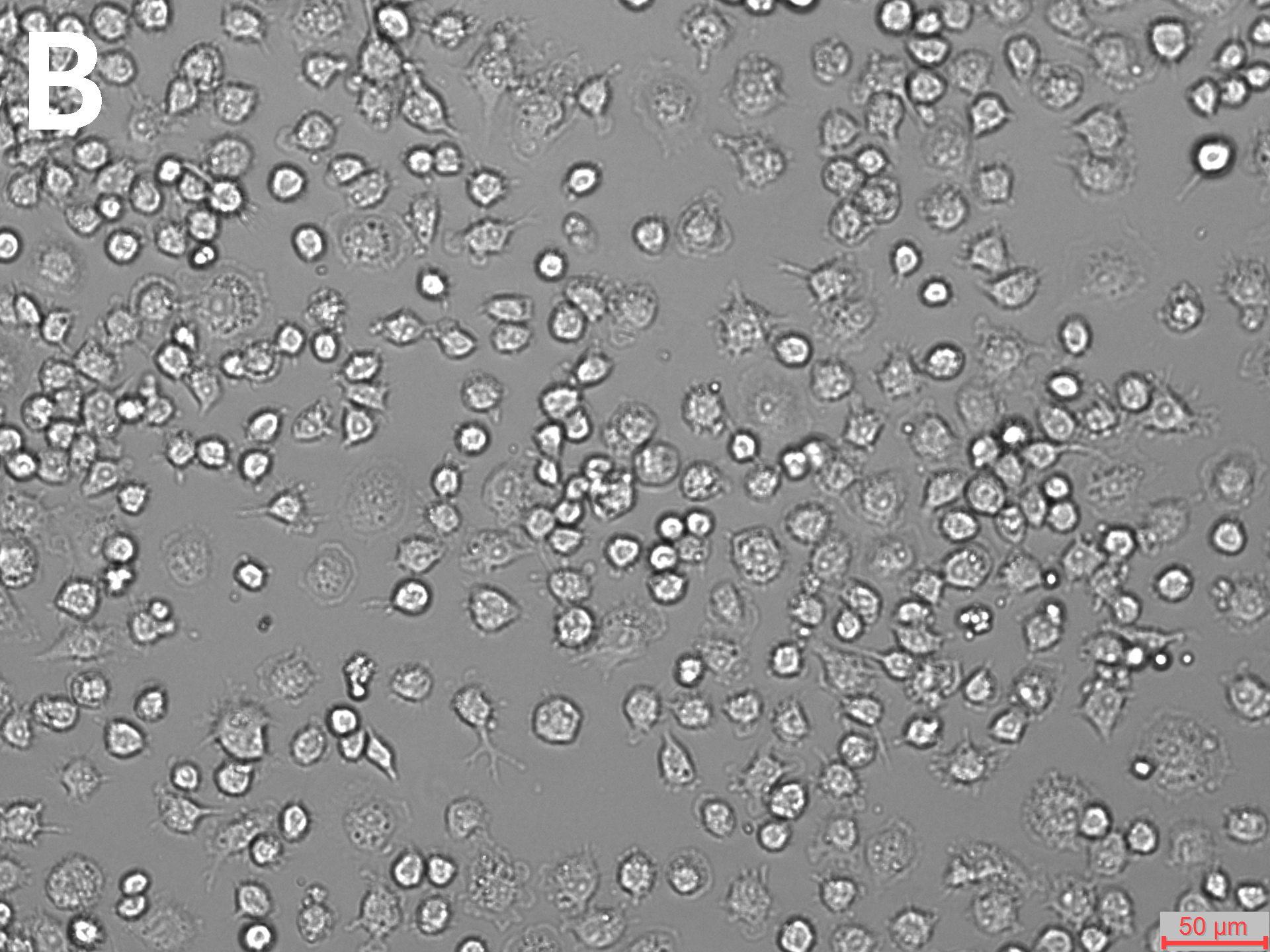

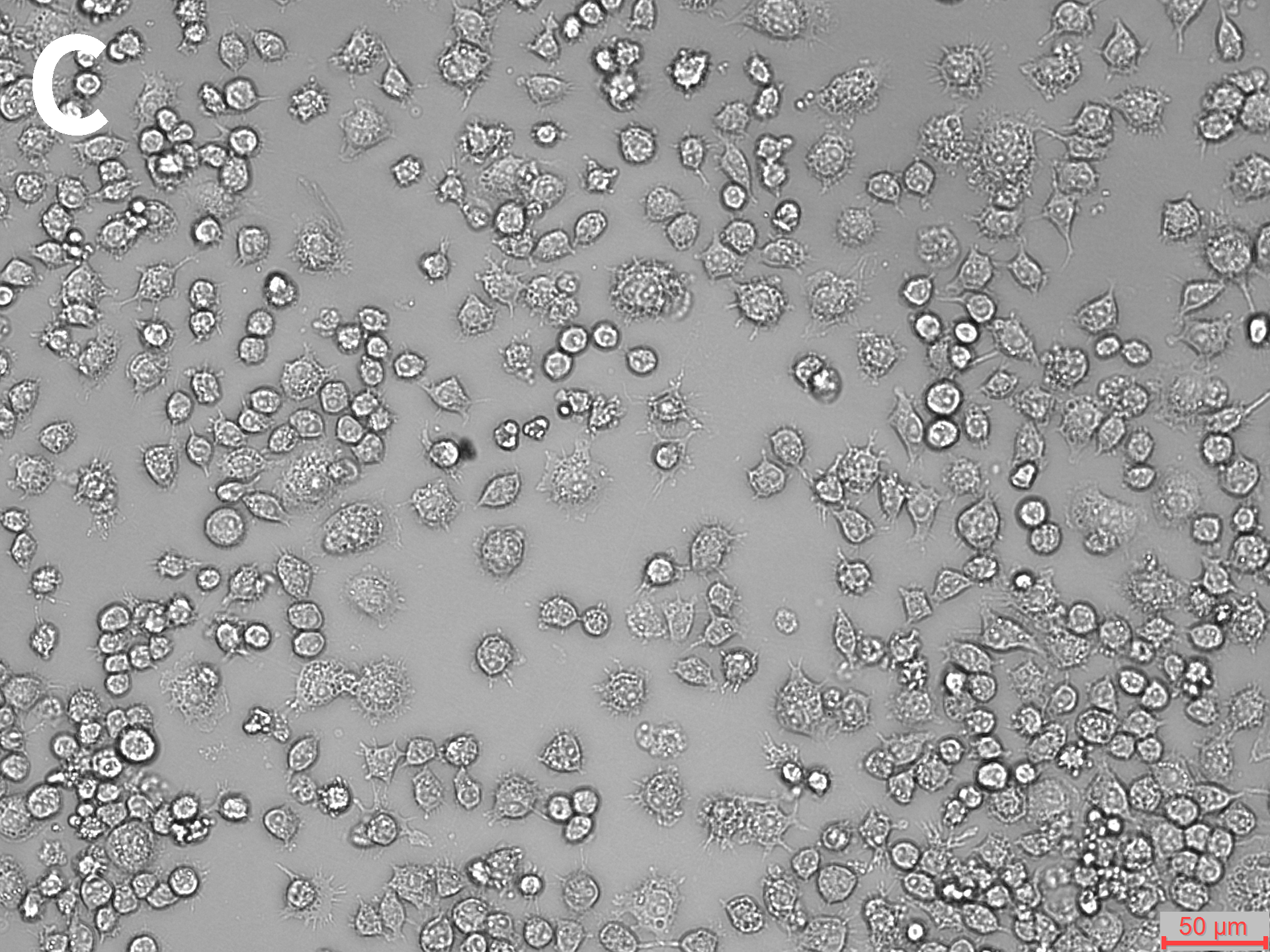

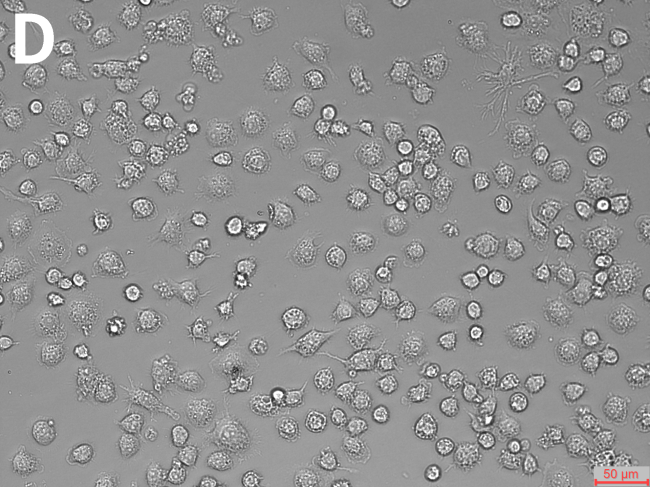

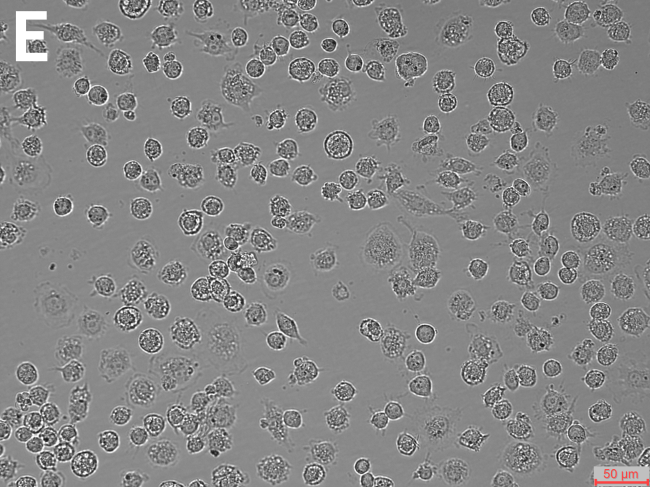


**Supplementary Fig S1**: RAW264.7 macrophage morphology. (A) Control, (B) EVs only, (C) LPS only, (D) EVs (10 μg/mL, 12 h) + LPS (1 μg/mL, 12 h), (E) EVs (20 μg/mL, 12 h) + LPS (1 μg/mL, 12 h).
